# Discovery of missing link between demosponges and hexactinellids confirms palaeontological model of sponge evolution

**DOI:** 10.1038/s41598-017-05604-6

**Published:** 2017-07-13

**Authors:** Joseph P. Botting, Yuandong Zhang, Lucy A. Muir

**Affiliations:** 1 Nanjing Institute of Geology and Palaeontology, 39 East Beijing Road, Nanjing, 210008 China; 2 Department of Natural Sciences, Amgueddfa Cymru – National Museum Wales, Cathays Park, Cardiff, CF10 3LP UK; 3 CAS Key Laboratory of Economic Stratigraphy and Palaeogeography, Nanjing Institute of Geology and Palaeontology, 39 East Beijing Road, Nanjing, 210008 China

## Abstract

The two major extant groups of siliceous sponges, Demospongiae and Hexactinellida, are generally regarded as sister groups forming the clade Silicea, although the nature of their last common ancestor is uncertain. The fossil record contains a diverse range of basal demosponges that appear to have evolved from hexactine-bearing reticulosan ancestors, although a compelling morphological intermediate has not previously been discovered. Here we describe a new species of fossil sponge, *Conciliospongia anjiensis* gen. et sp. nov., from the Late Ordovician (~444 Ma) Anji Biota of South China. This species has a reticulate, tufted skeleton of minute monaxon spicules, characteristic of the fossil demosponge family Hazeliidae and modern heteroscleromorphs, with hexactine spicules and a globose body form inherited from reticulosan ancestors. This transitional morphology had previously been hypothesized in palaeontological studies. This morphological intermediate between two extant classes further confirms siliceous sponge monophyly and demosponge–hexactinellid spicule homology, and supports the primitive, stem-silicean interpretation of simpler-structured fossil reticulosans.

## Introduction

Demosponges are the most diverse and abundant group of extant sponges^1, 2^, and have a long and abundant fossil record^3, 4^. This record, however, consists primarily of those groups with rigid, lithistid skeletons, or hypercalcified groups^4–6^. The skeleton of other demosponges is either primarily organic^1^, sometimes with some silica and/or aragonite biomineralization associated with chitinous fibres^7^, or consists of small siliceous spicules^1^ that are difficult to preserve articulated. These spicules can be relatively large in various groups of tetractine-bearing demosponges, but this appears to be a derived condition^8^; in the majority of Heteroscleromorpha^2^, such as the haplosclerids^9^, the primary spicule type is minute (sub-millimetre) monaxons, with even smaller microscleres.

The fossil record of non-lithistid, non-hypercalcified demosponges is relatively sparse^3, 4^. Most of the taxa from Cambrian rocks that have been traditionally described as protomonaxonid demosponges^3, 10^ are now recognized as belonging to a much earlier-branching group of sponges^11, 12^. Keratosan sponges have been described only occasionally, with the best-known examples being the family Vauxiidae, primarily from the Cambrian Burgess Shale-type faunas^12–15^. A few later Palaeozoic examples of keratosans are also known^16–18^, together with some agglutinating taxa that probably also fall into the Keratosa or Verongida^16, 19^. Spiculate demosponges have been much harder to identify and interpret accurately, and most early examples are included in the Hazeliidae^13, 14^. This family is characterized by minute monaxon spicules comprising a normally reticulating array of small overlapping tufts, and have been interpreted as basal crown-group demosponges^12^. In addition, an early Cambrian disarticulated heteroscleromorph has been described, showing the origin of their typical microscleres within the clade^20^; microscleres appear to have been absent from hazeliids.

**Figure 1:**
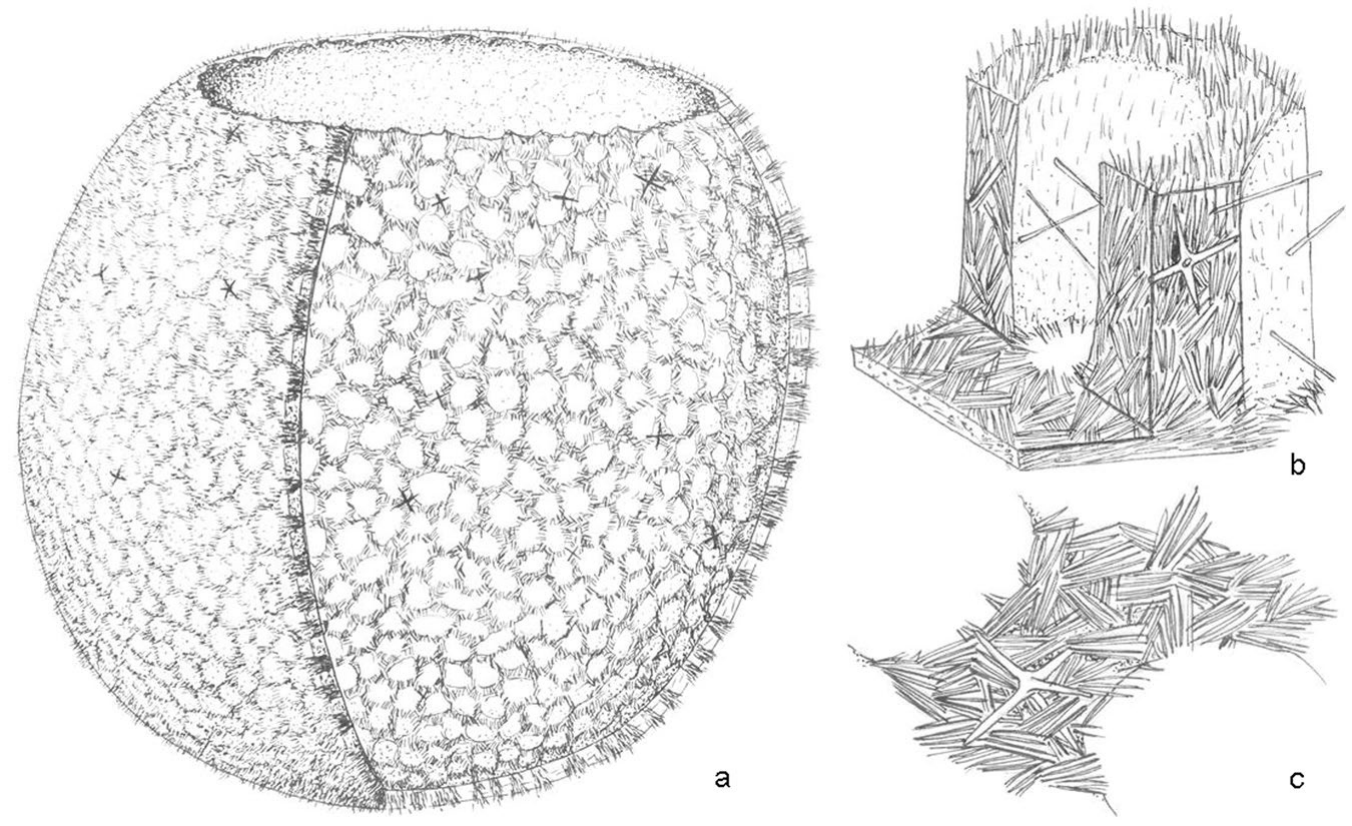
Reconstruction of *Conciliospongia anjiensis* gen. et sp. nov. (**a**) Overall cut-away view showing inner part of wall (right half) and outer surface (left half), with gaps in skeletal wall probably representing sites of choanocyte chambers, bounded by soft tissue walls; (**b**) three-dimensional cut-away reconstruction through wall thickness, illustrating tufts of fine monaxon spicules (up to 0.3 mm long), becoming more perpendicular to wall at exterior (upper) surface; (**c**) detail illustrating structure of inner part of wall. Sponge is approximately 35 mm wide.

**Figure 2:**
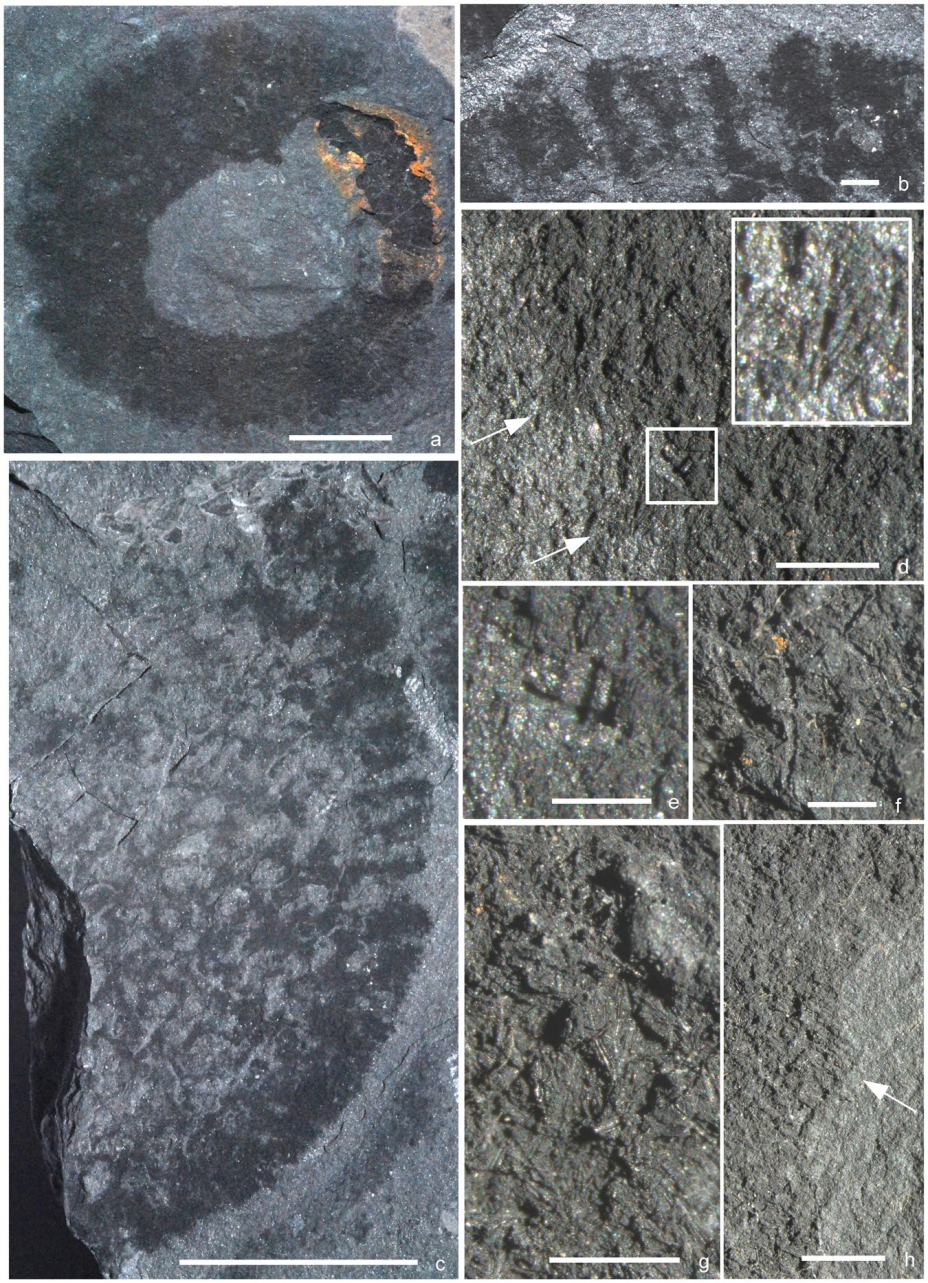
*Conciliospongia anjiensis* gen. et sp. nov. (**a**,** f–h**) Paratype (specimen number NIGP 165187): (**a**) Overall view of dorso-ventrally flattened specimen with osculum central; (**f**) detail from near oscular margin, including two large hexactines amongst fine monaxons; (**g**) detail of central upper part of body wall, showing tufts of fine parallel monaxons; (**h**) oscular margin showing cuspate margin (arrow) with localized projecting spicules, and tufted texture of wall, composed of small overlapping clusters of parallel monaxons (adjacent to arrowed cusp). (**b–e**) Holotype (specimen number NIGP 165186): (**b**) Detail of upper right margin, rotated anticlockwise with respect to (**c**), showing walls composed of spicule tufts and occasional spicules crossing spaces of reticulation; (**c**) overall view of partial, laterally-compressed specimen with well preserved reticulate skeletal wall; (**d**) detail of the lower right region of (**c**) on the counterpart, showing lateral projecting spicule tufts (arrowed, with lower arrowed region magnified and inset), position of (**e**) (box), and finely-tufted skeleton; (**e**) detail of (**d**), showing hexactine embedded in body wall. Scale bars: (**a**,** c**) 10 mm; (**b**,** d**,** h**) 1 mm; (**e**–**g**) 0.5 mm.

In molecular phylogenetics, there is now a general consensus on the sister-group arrangement of demosponges and hexactinellids, even when sponges have been found to be paraphyletic overall^21–23^, including some acceptance of the likely homology of siliceous spicules between the two classes^24^. Other topologies, however, are still being occasionally recovered^25^. The explicit question of homology of spicules between demosponges and hexactinellids is normally avoided, although significant differences in secretion mechanism and structure have been noted^26, 27^. Molecular analysis^8^ left open the question of whether demosponge skeletons were primitively spicular or organic, but palaeontological revision of the hazeliids and vauxiids^12^ implies spiculate ancestors, with spicules also present in basal vauxiids; the presence of silica in vauxiids has since been confirmed^15^. Furthermore, a study of spicule structure in the Ordovician hexactine-bearing reticulosan *Cyathophycus* ^28^ demonstrated hexagonal axial canal symmetry diagnostic of demosponges. This supports a speculative earlier suggestion^29^ that demosponges may have arisen from a sponge similar to *Cyathophycus* through loss of the external hexactine layer and exaggeration of the inner layer of fine monaxons. The new Ordovician sponge described herein (Figs 1 and 2) from the recently-discovered Anji Biota^30^ exhibits a previously unknown combination of characters that was predicted by that model.

## Results

### Systematic Palaeontology

Silicea Gray, 1867 (ref. 31)

Class, order and family uncertain

#### Remarks

The phylogenetic position of the new sponge can be constrained to the later part of the stem group of Demospongiae. This falls within Silicea, but no other less-inclusive clade, and therefore no further higher taxonomy is currently available.

### Genus *Conciliospongia* nov

#### Etymology

From Latin *conciliare*, to unite or bring together, referring to the demonstration of skeletal homology between reticulosans and demosponges.

#### Diagnosis

Globose, moderately thin-walled sponge with reticulate primary skeletal wall composed of small tufts of fine monaxons, becoming perpendicular to wall in outer part; also with isolated, robust, regular hexactines.

*Conciliospongia anjiensis* sp. nov.

#### Etymology

For Anji County, Zhejiang Province, China; the type locality.

Diagnosis. As for genus.

#### Holotype

NIGP 165186, from the latest Ordovician *Metabolograptus persculptus* Biozone of the Wenchang Formation, locality Zhuwukou, Anji County, Zhejiang, China; partial but well-preserved specimen in lateral view.

#### Paratype

NIGP 165187, from the type locality; complete, vertically compressed specimen.

#### Description

Bowl-shaped to globose sponge, 35 mm diameter in type material, with reduced osculum up to 20 mm in diameter. Height estimated at around 25–30 mm. Both specimens pyritized and partly weathered to black oxide minerals that retain clear impressions of spicules.

Body wall coarsely reticulate (Fig. 2b, c), with majority of gaps 0.8–1.2 mm across; intervening spicule tracts variable in width, but normally in the range 0.5–1.0 mm. In lateral view, outer region columnar, showing wall thickness of 1–2 mm. Reticulation broader and more open in upper part of holotype (Fig. 2c), and less clearly visible in paratype (due largely to different orientation of compression, but perhaps also to more massive pyritization).

Monaxon spicule tracts composed of irregularly oriented tufts of spicules up to 0.2 mm wide (Fig. 2d, g, h). Tufts become more parallel close to compressed lateral margin, especially in columnar region, implying that tufts are not wall-parallel, but irregularly oriented in three dimensions; this is also visible internally (Fig. 2g), with variable orientation of spicules inclined to plane of body wall. Thickness of marginal, columnar-appearing zone (Fig. 2b) shows that tufts are superimposed vertically as well as laterally overlapping, such that several tufts occur through the wall thickness. At surface, tufts project as bundles of outwardly-directed spicules (Fig. 2d). Regions between skeletal tracts presumed to have been composed dominantly of soft tissues with a central choanocyte chamber, but are partly or entirely crossed by occasional long, fine monaxon spicules up to 0.6 mm long (Fig. 2b), oriented mainly tangentially (but sometimes somewhat inclined) to the body wall. At lateral margins, these occasional cross-spicules are visible through virtually full thickness of wall.

Individual monaxons (Fig. 2g) densely packed and very fine, and up to 0.3 mm long; axial canals often visible as internal pyrite moulds, but not characterizable. Hexactines (Fig. 2e, f) present through body wall, particularly in outer part, as rare isolated spicules. More frequent in paratype, especially in upper area close to osculum (but not at the precise margin, or acting as marginalia). No regular orientation discernible, and spicules are widely separated and do not appear to be aligned with each other. Hexactines are robust, and mostly short-rayed, with majority having ray length up to 0.4 mm and basal diameter 0.05–0.10 mm; rays are straight and evenly tapered. Slightly larger spicules (Fig. 2f), with similar diameter but ray length up to 0.7 mm, are present in upper region of paratype.

Oscular margin not distinct in spiculation, but with weakly cuspate margin (Fig. 2h); no distinct marginalia visible, but some body wall monaxon tufts project inwards from margin. Basal region not certainly seen, but may be visible in holotype; no basalia observed. No microscleres visible.

## Discussion

The skeletal architecture of this monaxon-based sponge is effectively identical to that of a Cambrian hazeliid demosponge. The three-dimensional wall is more complex than that seen in *Crumillospongia* ^13^, but equivalent to that of more derived hazeliids such as *Hazelia lobata* ^14^ or *H*. *delicatula* ^32^, as reconstructed by Botting *et al*.^12^. The body form, however, is much more primitive, closely resembling the reticulate, globose body of *Crumillospongia* ^13^. In most *Hazelia* species the body form has evolved to become branching, lobate or palmate, but this is only very rarely seen among reticulosans^10^. The related Vauxiidae show similar reticulate wall architecture, but have strongly developed organic skeletons, reduced or absent monaxon spicules, and usually have an erect, branching body form^12, 14^.

The unique feature of *Conciliospongia anjiensis* gen. et sp. nov. is the presence of hexactines in combination with the monaxon-based skeleton. The hexactine morphology is typical of a wide range of reticulosan sponges, although the thinnest-walled reticulosans tend to have more slender-rayed spicules. Similar moderately robust hexactines to those in the new sponge form the dominant skeletal framework in the globose *Cyathophycus loydelli* Botting^33^, an advanced reticulosan with a regular spicule grid and an inner skeletal layer of monaxon spicules^29, 33^ and with rounded parietal gaps^28^. A similar secondary layer of obscure spicules in other species of *Cyathophycus* has been previously discussed^34^, also with rounded gaps through the secondary wall. The spicule microstructure of *C*. *loydelli* was considered to be an indicator of a close relationship to the demosponge stem lineage^28^, which would then have progressed through loss of hexactines to leave the architecturally flexible inner skeletal layer, which is composed of tracts or tufts of fine monaxons. Until now, however, no intermediates within this sequence of hexactine loss have been described, and the hypothesis has remained speculative.

The new species precisely fills this gap in the theoretical sequence. The globose body form is identical to that of *C*. *loydelli* and *Crumillospongia*, and shows none of the architectural complexity of the tubular, frequently branching vauxiids, or the more diverse morphologies seen in *Hazelia* ^14^. The tufted arrangement of fine monaxons is typical of the more advanced hazeliids, but easily derived from the tufted, semi-tractose structure seen in *Crumillospongia*, which is the last genus in the sequence to show a simple, globose body form. This in turn forms the basis for the skeletal architecture of extant heteroscleromorphs such as the Haplosclerida^10, 12^. The presence of low numbers of hexactines, which are absent from all fossil and recent demosponges, illustrates incomplete loss of the primary skeletal layer of its reticulosan ancestors. Their positions embedded within the perfectly articulated skeletal wall prove that the spicules have not been fortuitously superimposed, and their consistent morphology, intact condition and low density preclude deliberate incorporation of detrital spicules by an agglutinating sponge. The new species therefore represents a perfect evolutionary intermediate between a *Cyathophycus*-like reticulosan and a globose proto-demosponge like *Crumillospongia* (in which hexactines have been entirely lost). *Conciliospongia* therefore illustrates the stem group of demosponges, at a stage prior to the complete loss of the primary symplesiomorphies (hexactines, globose body form, thin wall) and also before the origination of shared demosponge characters (morphological plasticity, organic framework skeleton).

**Figure 3:**
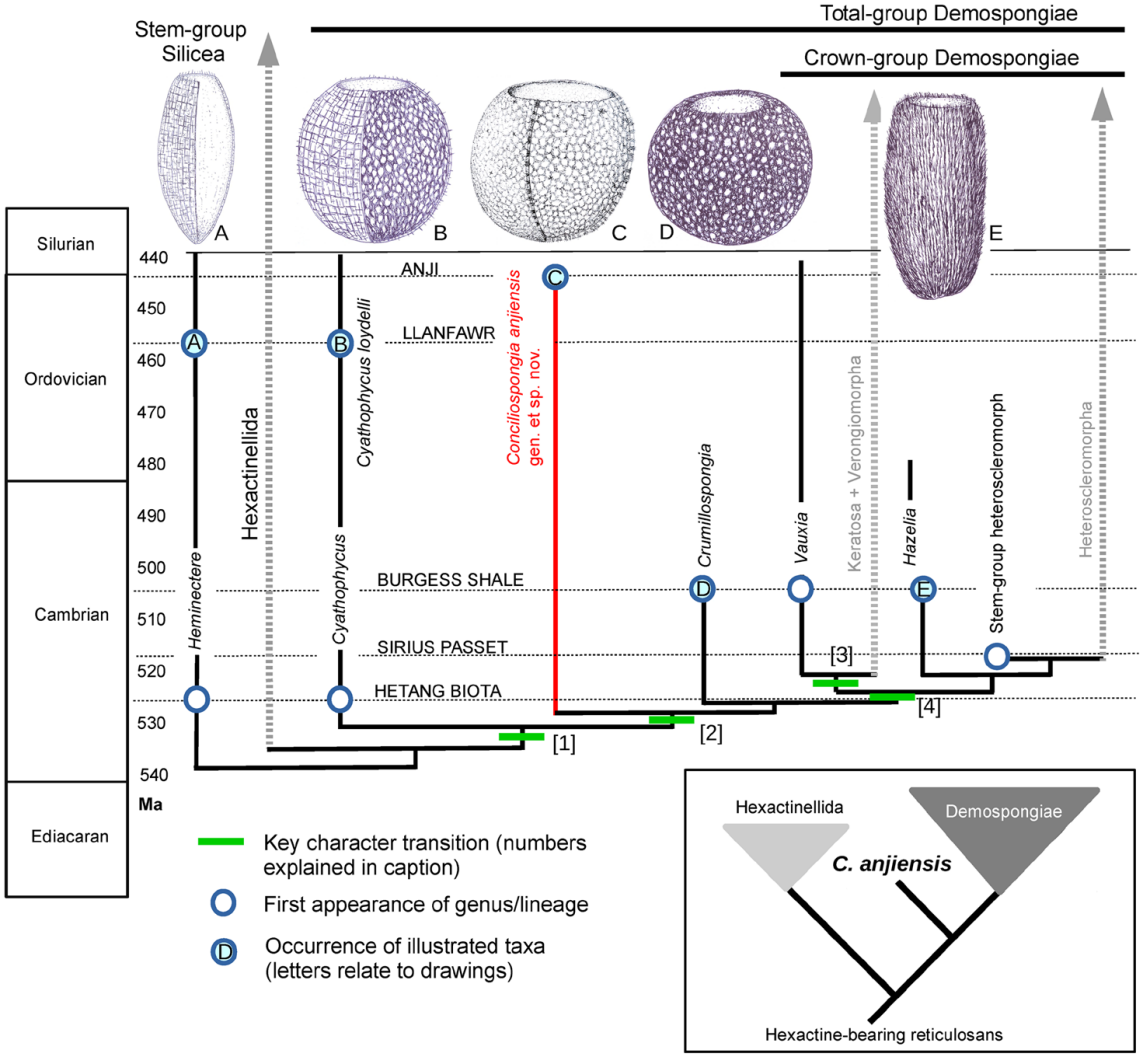
Summary of early demosponge evolution. Schematic diagram showing the phylogeny and stratigraphic distribution of the stem and basal crown groups of Demospongiae, including key fossil taxa and their occurrences. The inset summary shows the simplified position of *Conciliospongia anjiensis* gen. et sp. nov. relative to the extant silicean classes. Phylogenetic topology of extant taxa (thick dashed lines) conforms with molecular interpretations^8^. Primary hypothesis of fossil relationships are based on ref. 12 for vauxiid and hazeliid relationships and ref. 28 for *Cyathophycus loydelli*. Stem-group heteroscleromorph refers to ref. 20. The key skeletal transitions are the origination of a monaxon-based inner skeletal layer in *Cyathophycus* [1], subsequent loss of hexactines in the stem lineage of demosponges [2], and ultimately also the loss of monaxons within the Vauxiidae (leading to Keratosa and Verongiomorpha) [3]; the loss of a simple globose body form (and concomitant change in growth patterns) accompanied the origin of crown-group demosponges [4]. Cambrian occurrences of *Heminectere* (listed as *Protospongia* cf. *conica*) and *Cyathophycus* (described as *Diagoniella cyathiformis*) are after ref. 41, Ordovician occurrences of *Heminectere* and *Cyathophycus* are from ref. 33, and first appearances of *Crumillospongia*, *Vauxia* and *Hazelia* are from ref. 14.

The fossil record of sponges is notoriously incomplete, and important discoveries can emerge from surprising localities. The Anji Biota^30^ is relatively late (Late Ordovician) in comparison with previously-described early Cambrian crown-group demosponges^12, 20^, indicating a significant period of evolution since the separation of this sponge from the lineage leading to the base of the demosponge crown group (Fig. 3). This interval allowed the evolution of the relatively derived, three-dimensional wall structure in comparison with that of the thinner wall of *Cyathophycus* or *Crumillospongia*. Nonetheless, the body form remains as conservative as in reticulosans, suggesting that the biological or genetic innovations that allowed demosponge morphological plasticity to develop within the Hazeliidae had not yet occurred at this point in the lineage.

This discovery is of critical importance in demonstrating the evolutionary origin of demosponges, but as a result, has additional implications for interpretation of other reticulosan sponge fossils. The phylogenetic scenario that predicted this intermediate also requires stem-group status for many other sponge groups^28^. In particular, reticulosans that fall below *Cyathophycus* in structural complexity, such as *Protospongia* Salter^35^, *Heminectere* Botting^33^ and *Asthenospongia* Rigby, King and Gunther^36^ probably occupied phylogenetic positions below crown-group Silicea; this general evolutionary progression of wall complexity within sponges is widely recognized^14^, with the more simple globose, thin-walled architecture also shared with basal members of stem-group calcareans^37^. Confirmation of basal demosponge evolution at least requires a critical reassessment of basal sponge relationships based on these fossils, including palaeontological evidence that even calcareous and siliceous spicules are homologous, in the form of biminerallic spicules among both early calcarean-like^37, 38^ and protomonaxonid^39^ sponges. This scenario conflicts with traditional understanding based on the differences in secretion mechanisms of calcareous and siliceous spicules^27, 40^ and the lack of a model for transforming the spicule composition. The current discovery regarding demosponge origins and the homology of silicean spicules emphasizes the need for further detailed palaeontological studies, in addition to molecular and embryological work, to clarify sponge evolution.

## Material and Methods

The studied specimens were recovered as part of a diverse sponge fauna from the Upper Ordovician Wenchang Formation in Zhejiang Province, China^30^. The Wenchang Formation consists mostly of sandstone with an approximately ten-metre-thick mudstone interval in the middle-upper part, which has yielded articulated sponges at several sites. The new species was discovered at locality Zhuwukou (GPS coordinates 30°30.507′N, 119°22.712′E), a roadside section near the Fushi Reservoir, Anji County. The material can be dated as late *Metabolograptus persculptus* Biozone (Hirnantian, Late Ordovician, ~444 Ma) on the basis of graptolite biostratigraphy^30^. Material was photographed using a Zeiss Discovery V.20 stereomicroscope and Axiocam 506 camera system (photomicrographs), or with Nikon D80 with extension tubes and 105 mm macro lens (wider-angle photographs). Specimens are deposited in the Nanjing Institute of Geology and Palaeontology (NIGP).
